# Analysis of registered radiological equipment in Kenya

**DOI:** 10.11604/pamj.2021.40.205.29570

**Published:** 2021-12-06

**Authors:** Lynne Muthoni Gathuru, Gabriel Daniel Onditi Elias, Richard Denys Pitcher

**Affiliations:** 1 Department of Radiology and Imaging, School of Medicine, Moi University, Eldoret, Kenya,; 2 Department of Medical Imaging and Clinical Oncology, Faculty of Medicine and Health Sciences, Stellenbosch University and Tygerberg Academic Hospital, Cape Town, South Africa

**Keywords:** Diagnostic radiology, healthcare, Kenya

## Abstract

**Introduction:**

diagnostic radiology plays a key role in healthcare. Proper planning of healthcare requires accurate and robust data. There´s, however, paucity of comprehensive figures on radiological equipment in the African setting. The goal of this study was to carry out an in-depth analysis of the registered radiological equipment in Kenya, a lower middle-income African country, and compare the findings to published international data.

**Methods:**

data on radiological equipment were obtained from the Kenya Nuclear Regulatory Authority and analyzed as units/million of the population by imaging modality, health service sector and administrative units. The findings were then compared to published international data.

**Results:**

there has been an overall increase in the number of radiological equipment in comparison to data published in 2013, with a relatively uniform distribution of resources across all eight regions. General radiography is the most available modality at 24.5 units/million with the majority of the equipment owned privately, while the public sector (9.6 units/million) has less than a half of the WHO recommendation of 20 units/million. Accessibility to computerized tomography (CT) scan, fluoroscopy and mammography in the public sector closely mirrors that of South Africa. On the contrary, positron emission tomography-computerized tomography (PET-CT) is the least-resourced modality and is currently only available in the private sector.

**Conclusion:**

the increased number and homogenous distribution of radiological resources can largely be attributed to the Managed Equipment Services project launched by the national government in 2016. More needs to be done with regards to availability of PET/CT scanners and general radiography equipment in the public sector.

## Introduction

Health care is a significant part of any country´s economy with the accessibility to quality healthcare being fundamental to economic growth. Provision of health-care services, and safeguarding the well-being of the population is recognized in the sustainable development goals (SDGs) as crucial in ensuring the continued development of a country [1].

Diagnostic imaging forms a key component in modern health care, playing a vital role in the diagnosis and prognostication of disease as well as in monitoring response to treatment [2]. The number of radiological examinations performed has increased over the past few years. This has been attributed to technological advances in imaging, increasing availability of imaging facilities, and a rising demand by both patients and health care workers [3]. The surge in the use of diagnostic imaging has been associated with increasing costs and exposure to radiation. However, despite radiological equipment being expensive and the high costs of radiological examinations, diagnostic imaging is of great value in individual patient management and health care systems as a whole [4]. In addition, radiological services are integral in the provision of primary health care [5,6].

Huge disparities exist in the availability of radiology services between the developing and developed world. An estimated 50-67% of the world´s population has no access to basic radiological facilities, with the majority living in the rural areas of low- and middle- income countries (LMIC) [7,8]. This is in stark comparison to high-income countries where state-of-the-art radiological equipment are readily available with risk of overutilization [8].

Furthermore, differences exist within developing countries with the private health facilities being better equipped than those in the public sector [9]. Access to private facilities for most people is, however, challenging, owing to high cost, lack of proper health insurance cover, and location in urban areas [10]. In Kenya, the bulk of the population (72.5%) lives in rural areas [11], with only approximately a fifth (19.59%) of Kenyans having health insurance. Out-of-pocket expenditure on healthcare has been shown to have dire effects on households, particularly in rural areas and among the poor [12].

It is in view of the above and in line with the goal to achieve Universal Health Coverage (UHC) by the year 2030 as per the SDGs, that the government of Kenya embarked on a scheme known as the Managed Equipment Services (MES) to ensure equitable distribution of various facilities including radiological equipment throughout the country. The constitution of Kenya enshrines a devolved system of government with two levels: the national government and county governments (47 in number). MES is a public-private partnership between the national government, county governments and private companies that kicked off in 2016, and has led to the equipping of 98 hospitals countrywide (2 in each county and 4 national referral hospitals) with modern medical equipment including diagnostic imaging facilities [13].

Proper planning of health care including diagnostic imaging requires accurate, comprehensive and robust data. The United Nations (UN) in 2007 adopted resolution 60.29 in the Sixtieth World Health Assembly, urging member countries to “to collect, verify, update and exchange information on health technologies in particular medical devices as an aid to their prioritization of needs and allocation of resources” [14]. Despite the fact that the World Health Organization (WHO) has published data on the diagnostic imaging equipment in member states, the figures are based mainly on country surveys, which run the risk of not being up-to-date and excludes information on basic radiological equipment such as general radiography [15]. In the African context, there is paucity of data on radiological equipment with the published figures currently available being from South Africa, Tanzania, Zimbabwe, Zambia and Uganda. This study aims to provide a comprehensive analysis of the registered radiological equipment available in Kenya, a lower middle-income African country, and compare this to available international data. This will not only provide baseline information for use in planning for health care but will also go a long way in assessing the imaging capacity of the country, and contribute to the discourse on the minimum imaging capability required in a population.

## Methods

**Study design and setting:** this was a cross-sectional descriptive study conducted in January 2020 in Kenya, which is a lower middle-income East African country covering an area of 580,876 km^2^ with a population of 47.6 million people according to the 2019 National Census [16]. The gross domestic product (GDP) of Kenya in 2019 was 95.5 billion US dollars [17]. An estimated 5.2% of the GDP was spent on healthcare in 2018 [18] with 19.59% of Kenyans having health insurance [19].

**Data collection:** data on the number of registered diagnostic radiology units including general radiography, computerized tomography (CT) scan, fluoroscopy, mammography and PET/CT were obtained from the Kenya Nuclear Regulatory Authority (KNRA), and information on the population of Kenya from the results of the 2019 national census [16].

**Data analysis:** the imaging capacity of the country was analyzed in terms of number of diagnostic radiology units per million population by imaging modality, healthcare sector (public and private) and administrative units (regions and counties). For this analysis, the former provinces of Kenya were considered as regions namely Coast, North Eastern, Eastern, Central, Rift Valley, Western, Nyanza and Nairobi. The findings were then compared to international data including figures from South Africa, Zambia, Zimbabwe, Tanzania and Uganda, as well as data from member countries of the Organization of Economic Development (OECD). Established in 1961, OECD is an intergovernmental organization whose goal to foster economic development in addition to social and environmental progress. It currently constitutes 37 member nations, majority of whom are high-income countries.

**Ethical approval:** approval to conduct the study was obtained from the National Commission for Science, Research and Innovation (NACOSTI), permit no: NACOSTI/P/19/60137/28062.

## Results

**Figure 1:**
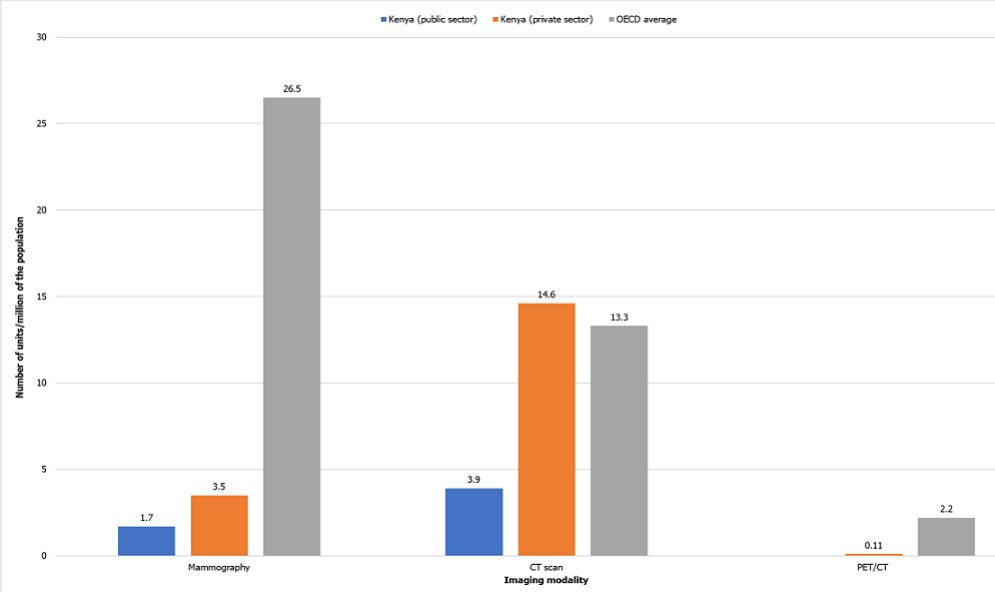
comparison of radiological equipment in Kenya (public and private sectors) and OECD average

Kenya´s resources in diagnostic imaging are shown in Table 1. Comparisons of radiological resources as well as health, demographic and economic indicators in Kenya, Tanzania, Zimbabwe, South Africa, Zambia, Uganda and OECD countries are illustrated in Table 2, Table 3 and Figure 1.

**Table 1:** Radiological equipment in Kenya

Region(Mil. Pop)^§^	PopulationDensity*	General Radiography			CT scan			Fluoroscopy			Mammography			PET/CT		
		Private	Public	Total	Private	Public	Total	Private	Public	Total	Private	Public	Total	Private	Public	Total
**Coast(4.3)**	52.6	104.9	15.2	32.8	11.8	1.7	3.7	10.6	4.9	6.0	5.9	2.0	2.8	0	0	0
**North Eastern(2.5)**	19.5	18.4	4.5	7.2	6.1	1.5	2.4	0	1.5	1.2	0	1.0	0.8	0	0	0
**Eastern(6.5)**	44.5	46.4	8.9	16.3	7.5	1.5	2.6	2.2	1.8	1.9	0.7	1.5	1.3	0	0	0
**Central(5.5)**	416.8	93.1	10.9	27.0	17.7	1.4	4.6	16.8	3.6	6.2	4.7	1.4	2.0	0	0	0
**Rift Valley(12.8)**	69.7	65.2	10.0	20.9	8.8	1.3	2.7	3.6	2.1	2.4	2.4	1.7	1.8	0	0	0
**Western(5.0)**	604.8	21.3	6.9	9.8	5.1	1.5	2.2	0	0.2	0.2	1.0	0.5	0.6	0	0	0
**Nyanza(6.3)**	497.5	44.8	6.1	13.7	10.6	1.4	3.2	4.9	0.6	1.4	0.8	1.2	1.1	0	0	0
**Nairobi(4.4)**	6246.7	352.9	11.0	78.0	62.7	0.6	65.0	46.4	2.3	10.9	16.3	0.6	3.6	1.16	0	0.23
**Total(47.6)**	81.9	86.2	9.4	**24.5**	14.6	1.3	**3.9**	9.1	2.1	**3.5**	3.5	1.3	**1.7**	0.11	0	**0.02**
**Public :Private**		1 : 9.2			1 :11.2			1 : 4.3			1 : 2.7			N/A		
**Lowest:highest**		19.2	3.4	10.8	12.3	2.8	29.5	N/A	24.5	54.5	N/A	4	6	N/A	0	N/A

CT: Computed Tomography, PET: Positron Emission Tomography

**Table 2:** comparison of registered radiological equipment by modality and health service sector in Kenya, Tanzania, Zimbabwe, South Africa, Zambia and Uganda

Modality		Kenya	Tanzania	Zimbabwe	South Africa	Zambia	Uganda
		units per million population					
**General radiography**	Public	9.4	5.66	11	19.8	11.5	3.1
	Private	86.2	25.89	160	104.0	82	197.5
	Total	24.5	9.02	26	34.8	14.3	12.8
	Public:private	1: 9.2	1: 5	1: 16	1: 5.3	1: 7.1	1: 63.7
	Lowest: highest (public)	1: 3.4	1: 2.3	1:5	1:2.6	1: 3.1	1: 2.2
**CT scan**	Public	1.3	0.08	0.6	1.7	0.51	0.2
	Private	14.6	2.15	9	20.7	7.65	0.4
	Total	3.9	0.42	1.5	5.0	0.79	0.6
	Public: private	1: 11.2	1: 27	1:2	1: 12.1	1: 15	1:2
	Lowest: highest (public)	1: 2.8	N/A	N/A	1: 6.8	N/A	N/A
**Fluoroscopy**	Public	2.1	0.83	0.1	2.5	0.57	0.3
	Private	9.1	1.88	7	26.8	-	0.6
	Total	3.5	1.0	0.8	6.6	0.55	0.8
	Public: private	1: 4.3	1:2	1: 69	1: 10.7	N/A	1:2
	Lowest: highest (public)	1:24.5	1: 2	N/A	1: 8.6	N/A	1: 2.5
**Mammography**	Public	1.3	0.24	0.2	1.29	1.02	0.1
	Private	3.5	0.67	6.1	22.3	6.1	0.4
	Total	1.7	0.31	0.8	4.96	1.22	0.5
	Public: private	1: 2.7	1:3	1: 3	1: 17.3	1: 5.98	1:4
	Lowest: highest (public)	1: 4	N/A	N/A	N/A	1: 3.9	N/A
**PET/CT**	Public	0	-	-	0.08	-	-
	Private	0.11	-	-	0.59	-	-
	Total	0.02	-	-	0.16	-	-
	Public: private	N/A	-	-	1: 7.37	-	-
	Lowest: highest (public)	0	-	-	N/A	-	-

CT: computed tomography, PET: positron emission tomography

**Table 3:** comparison of indicators in Kenya, Tanzania, Zimbabwe, South Africa, Zambia, Uganda, and OECD countries

	Kenya	Tanzania	Zimbabwe	South Africa	Zambia	Uganda	OECD countries
**Demographic indicators**							
Population (X 106 )	47.6	44.9	12.9	58.8	17.9	40.3	1,360
Area (1000 km2)	580.37	947.3	390.76	1219.09	752.61	241.55	37,607.70
Population annual growth rate (%) 2019	2.27	2.95	1.42	1.34	2.89	3.56	0.54
**Health and economic indicators**							
GDP (in billion USD) 2019	95.5	63.2	21.4	351.4	23.3	35.2	53,699
Annual GDP growth rate (%) 2019	5.4	5.8	-8.1	0.2	1.4	6.8	1.6
Health Expenditure per Capita (USD) 2018	88.39	36.82	140.32	525.95	75.99	43.14	4,899.63
Total health expenditure as % of GDP (2018)	5.2	3.6	4.7	8.3	4.9	6.5	12.5
Health Insurance (% of total population insured)	19.6	16	10	17	4	5	
Out-of-pocket health expenditure (as % of total health expenditure) 2018	23.62	23.98	24.36	7.73	9.98	38.38	13.7

**Kenya country data:** overall, general radiography is the most accessible imaging modality at a total of 24.5 units per million of the population, while PET/CT was the least accessible at 0.02 units. Nairobi is the only region with the full range of imaging modalities as well as the highest total number of units per million of the population for each modality countrywide, followed by the Coast and Central regions which have the second and third highest resources. The bulk of the equipment in these areas is in the private sector. Despite being the second most densely populated region, Western is the least equipped having the lowest number of units across the board except for general radiography, where the North Eastern region is least equipped at 7.2 units per million of the population.

Fluoroscopy has the highest discrepancy in the total number of units between the least and the most equipped regions at 54.5-fold followed by CT scan at 29.5-fold. The total number of mammography units is homogenously low ranging from Western with 0.6 units/million of the population to Nairobi with 3.6 units. Most of the imaging modalities including general radiography, CT scan, fluoroscopy and mammography are available in the public sector in all eight regions with the distribution being relatively homogenous, except for PET/CT which is only available in the private sector in Nairobi. Computerized tomography scan has the greatest disparity in the public versus private health sectors at 11.2-fold more resources in the private sector.

**Comparison with international data:** Kenya has greater resources than Tanzania, Zimbabwe, Zambia and Uganda in the public sector across all imaging modalities, with the exception of general radiography where Zimbabwe and Zambia have approximately 1.2-fold more units. The imaging capacity in Kenya closely approximates that of South Africa for CT scan, fluoroscopy and mammography. This, however, does not apply for general radiography where Kenya has 2-times fewer units, and PET/CT where Kenya has no units in the public sector compared to 0.08 units in South Africa. The number of CT scan units in the private sector in Kenya is higher than the OECD average compared to that of the public sector which is 3.4-times lower. Accessibility to mammography and PET/CT is considerably inferior in both the Kenyan public and private sectors relative to the OECD average.

## Discussion

This study provides a comprehensive analysis of the diagnostic imaging equipment in Kenya, which would serve as robust baseline data for planning for health care at the national and county level. In addition, comparison is made to international data including figures from five African countries of different economic standing as well as data from OECD countries, hence contributing to the debate on the minimum radiological equipment required for a population.

The overall increase in the number of diagnostic radiology equipment in comparison to data published by Korir *et al*. in 2013 can, to a large extent, be attributed to the MES, a public-private partnership project (PPP) introduced in 2016, that has equally resulted in availability of more imaging modalities in the public sector with a relatively uniform distribution of resources across all eight regions. The study by Korir *et al*. focussed on quality management systems in radiology in Kenya, and indicated a total of 20, 0.8 and 0.5 units per million of the population for general radiography, CT scan and mammography respectively [20]. Greater investment by the private sector has also played a role in the surge of radiological facilities in the country [21] as evidenced in Table 1 which indicates that the lion´s share of the radiology resources is owned privately.

Accessibility to radiology resources, especially in the private sector, appears to be related to urbanization. This is evidenced by Nairobi, which is the capital city and main referral center of Kenya, being the only region with the full spectrum of imaging modalities and the highest total number of units for each modality countrywide. The Coast and Central are the second and third most-equipped regions respectively with the majority of the equipment in the Coast being located in Mombasa, the second-largest city in Kenya, while Central is situated in close proximity to Nairobi. Additionally, modalities including fluoroscopy and CT scan, which show the largest discrepancies between the least and most equipped regions, have the bulk of the equipment in the private sector in urban areas as illustrated in Table 1.

The inverse relationship of the availability of imaging modalities in the public sector depending on their cost per unit that had been demonstrated in previous studies [4,8] is reflected in this present study, where general radiography and fluoroscopy which are relatively lower-priced are more accessible when compared to more expensive equipment such as CT and PET/CT. This is, however, not true for the private sector where CT scan is the second most available resource after general radiography, with the number of CT scan units being 1.6- and 4.2 times higher than that of fluoroscopy and mammography respectively. The number of CT scan units in the private sector (14.6 units) is also relatively higher than the OECD average (13.3 units). Rising demand for CT scan could account for this variation [22] causing greater investment by private health care providers.

The imaging capacity of Kenya for CT scan, fluoroscopy and mammography in the public sector closely mirrors that of South Africa. Conversely, the number of general radiography units is significantly lower at 9.8, units compared to the WHO recommendation of 20 units per million of the population [8], and 2-times lower than South Africa. Increasing the number of diagnostic radiography units in primary health facilities in the rural areas would go a long way in mitigating this. The health service system in Kenya is organized into six main tiers: level 1 care is provided by community health workers at the community level and is mainly home-based. Dispensaries (level 2) and health centers (level 3) offer primary care from where patients may be referred to county referral hospitals which form the fourth and fifth tier. The highest level of referral is to national referral hospitals (level 6) [23]. Most Kenyans (72.5%) live in the rural areas [11] where the nearest primary health facilities are dispensaries and health centers, [24] majority of which lack basic radiological services such as ultrasound and X-ray. Shortage of well-trained radiology staff in these facilities also poses a great challenge. Kenya continues to have a high burden of diseases such as pulmonary TB [25] for which general radiography serves as a useful screening tool [26]. Stocking health centers in the rural areas with general radiography units and providing the required human resource would greatly improve health care in these regions.

PET/CT is the least-resourced imaging modality in Kenya being currently only available in the private health care sector. Similar findings are observed in sub-Saharan countries with Zimbabwe, Tanzania, Zambia and Uganda lacking PET/CT scanners, and PET/CT being the least available modality in South Africa in both public and private sectors at 0.08 and 0.59 units respectively [8,22,27]. This is in comparison to the OECD average at 2.2 units per million of the population. An economic challenge lies therein, with huge differences in GDP and percentage of GDP allocated to health care between OECD and sub-Saharan countries as illustrated in Table 3. Setting up and maintaining PET/CT scanners is capital-intensive [28] with further costs necessary to achieve the skilled personnel required to run the equipment [29]. However, in view of the growing burden of oncology [30], which is a main indication for PET/CT imaging [31], it is essential to install more PET/CT scanners in the country.

It is imperative to consider the minimum radiological equipment required in a population based not only on the numbers of equipment, but also on the human resource required to operate the equipment, as well as the economic strength and public health needs of the population. This is so as to ensure that the radiological resources are “appropriate, accessible and affordable” [32] and flawlessly integrated into the existing health systems. As a result, more work needs to be done to assess the radiological needs in individual lower middle-income countries.

**Strengths and limitations:** the strength of this quantitative study is its basis on the official Kenya Nuclear Regulatory Authority (KNRA) database of registered radiological equipment as well as comparison to published statistics from other countries. It is the sixth in a series of planned manuscripts analysing the radiological resources in individual African countries that is hoped to spark further interest in carrying out in-country audits of radiological equipment. A limitation of the study is the exclusion of data on ultrasound and Magnetic resonance imaging (MRI) equipment, which are presently not registered with KNRA. This is a common limitation for current analyses of national diagnostic imaging resources and provides a major challenge in assessing the full imaging capacity of low- and middle-income countries, especially with regards to ultrasound equipment, which forms a fundamental component of basic radiological resources. Registration of ultrasound and MRI equipment would go a long way in facilitating healthcare planning.

## Conclusion

The increased number and homogenous distribution of radiological resources in the public sector can largely be attributed to government efforts to equip hospitals through the Managed Equipment Services project. More needs to be done with regards to availability of PET/CT scanners and general radiography equipment in the public sector.

### What is known about this topic

- The vital role diagnostic radiology plays in healthcare in the diagnosis and prognostication of diseases as well as monitoring response to treatment;
- Paucity of comprehensive data on registered radiological equipment worldwide with the current published data in African countries from South Africa, Tanzania, Zimbabwe, Zambia and Uganda.

### What this study adds

- It provides a detailed analysis of the radiological resources in Kenya, demonstrating an overall homogenous distribution of equipment with deficits in the public sector with regards to general radiography and PET/CT as well as highlighting similarities and differences with published figures from other African countries and OECD member nations;
- It illustrates the importance of an in-country audit of radiological equipment which aids in planning for healthcare.
